# Manual of Physiology

**Published:** 1857-12

**Authors:** 


*Manual of Physiology.* By WILLIAM SENHOUSE KIRKES, M. D., Fellow of the Royal College of Physicians; Assistant Physician to, and Lecturer on Botany and Vegetable Physiology at, St. Bartholomew's Hospital. A new and revised American, from the last London edition, with two hundred illustrations. Philadelphia: Blanchard & Lea. 1857.

We received this work some months since, but have inadvertently omitted to notice it. It has been so long familiarly and favorably known to the profession, that it is unnecessary for us to further point out its merits. It has always been one of the most useful and convenient handbooks to the student, which we have in any department. This volume is considerably enlarged, and is published in the handsome style which characterizes all books coming from the house of Blanchard & Lea. F.

